# Chromosome engineering in zygotes with CRISPR/Cas9

**DOI:** 10.1002/dvg.22915

**Published:** 2016-01-25

**Authors:** Katharina Boroviak, Brendan Doe, Ruby Banerjee, Fengtang Yang, Allan Bradley

**Affiliations:** ^1^ Wellcome Trust Sanger InstituteWellcome Genome CampusHinxtonCB10 1SACambridgeUnited Kingdom

**Keywords:** CRISPR/Cas9, large structural variants, zygote injection

## Abstract

Deletions, duplications, and inversions of large genomic regions covering several genes are an important class of disease causing variants in humans. Modeling these structural variants in mice requires multistep processes in ES cells, which has limited their availability. Mutant mice containing small insertions, deletions, and single nucleotide polymorphisms can be reliably generated using CRISPR/Cas9 directly in mouse zygotes. Large structural variants can be generated using CRISPR/Cas9 in ES cells, but it has not been possible to generate these directly in zygotes. We now demonstrate the direct generation of deletions, duplications and inversions of up to one million base pairs by zygote injection. genesis 54:78–85, 2016. © 2016 The Authors. genesis Published by Wiley Periodicals, Inc.

## INTRODUCTION

Many human genetic disorders are caused by structural variants such as deletions, duplications, and inversions of genomic regions involving several genes. Many deletion syndromes caused by deletions spanning several million base pairs, have severe phenotypes, for example, DiGeorge and Smith–Magenis syndromes. Large duplications are somewhat more benign, unless the duplicated region is very large, such as that seen in Down's syndrome. Inversions are often asymptomatic because relatively few genes are affected, although very large heterozygous inversions can result in infertility.

Linkage orders of genes tend to be conserved between humans and mice over the size ranges of pathologic deletion and duplication syndromes in humans. This has facilitated the process of modeling chromosomal rearrangements involved in human disease in mice. The size of these alterations has precluded their generation in a single step, therefore they have been generated by a multistep process termed chromosome engineering involving two gene targeting steps followed by Cre‐*loxP* recombination (Ramirez‐Solis *et al*., 1995; Smith *et al*., 1995). The desired chromosomal alterations can be wholly generated in embryonic stem (ES) cells and subsequently established in mice, or the rearrangements may be generated by Cre‐*loxP* recombination between homologous chromosomes during meiosis (Herault *et al*., 1998). These experimental approaches have yielded a number of important mouse models of human deletion disorders such as DiGeorge (Lindsay *et al*., 1999) and Smith–Magenis syndrome (Walz *et al*., 2003). Duplication disorders have also been modeled such as a 6‐Mb duplication on Chr 15q11‐13 seen in autism (Nakatani *et al*., 2009). The very large Down's syndrome trisomy Chr21 was modeled by generating in ES cells duplications on three different mouse chromosomes corresponding to human chromosome 21 conserved linkage groups and crossing them into a single mouse line (Yu *et al*., 2010).

Despite these successes, the multistep processes required to generate these alleles has proven to be challenging for many laboratories and the new gene editing tools have offered solutions. Using zinc‐finger nucleases and transcription activator‐like effector nucleases (TALENS), inversions and duplications extending to several 100 kb in mammalian cell lines and up to 1 Mb in zebrafish have been generated (Gupta *et al*., 2013; Xiao *et al*., 2013). More recently, CRISPR/Cas9 technology has been used to create large structural rearrangements in several mammalian cell lines (Canver *et al*., 2014; Choi and Meyerson, 2014). This success has now been extended to ES cells in a process termed CRISVar to generate large deletions, duplications, and inversions of up to 1.6 Mb which were then used to establish one of these alleles in the germ line of mice (Kraft *et al*., 2015). The CRISVar process appears to be distance dependent, in the million base‐pair size range 1%–2% of ES cells clones could be isolated with deletions or inversions but clones with duplications were not identified.

In this study we have sought to extend the CRISVar process by exploring the feasibility of generating large structural variants directly by zygote injection of the CRISPR/Cas9 components. Although deletions of 65 kb have been generated by zygote injection previously (Zhang *et al*., 2015), this is relatively small and the relationship between size and efficiency was unknown. We also wished to establish a reliable method for generating large duplications. We demonstrate here that deletions and inversions of 1Mb can be efficiently generated by zygote injection of the CRISPR/Cas9 components. We also show that similar sized duplications can be generated, although at lower frequencies.

## RESULTS

In this study we targeted the region around the tyrosinase locus (*Tyr)* because the arrangements in this region should be non‐lethal and *Tyr* provides the ability to score re‐arrangements phenotypically (Deol *et al*., 1986; Le Fur *et al*., 1996; Mintz, 1967). The zygotes used in this study were C57BL6/N, thus homozygous loss‐of‐function mutations will be readily visible as albino fur (Supporting Information Fig. 1).

### Quad gRNAs and Bridging Oligonucleotides to Direct Small Deletions at the *Tyr* Locus

**Figure 1:**
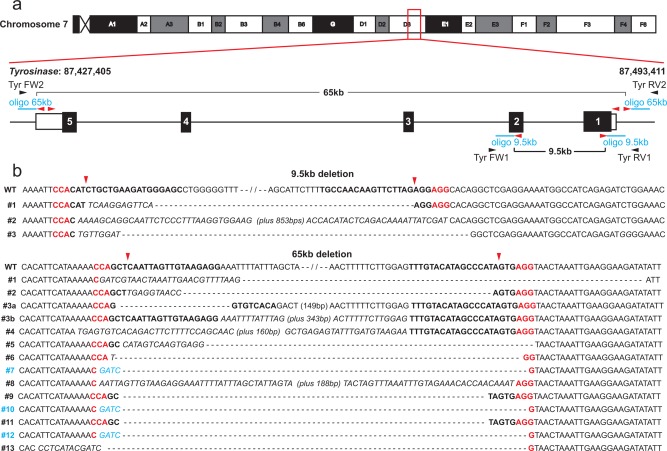
Generation of small deletions using CRISPR/Cas9. (**a**) Schematic representation of the CRISPR/Cas9 gRNA sites (*red arrowheads*) for the 9.5 and 65 kb deletions as well as genotyping primer sites (*black arrowheads*) and oligonucleotides (*blue*). (**b**) Deletion junction sequences for the 3 founders for the 9.5 kb deletion and the 13 founders for the 65 kb deletion with gRNA sites indicated in *bold* and PAM sites in *red*. The expected gRNA cut site is indicated by *red arrowheads*. Sequences marked in *blue* show deletions with defined breakpoints directed by the oligonucleotide.

To establish the system we co‐injected two gRNAs spanning 9.5 kb which target exons 1 and 2 of the *Tyr* gene (Fig. 1). These injections also included an oligonucleotide which was designed to bridge the deleted region. One third of the mice born were phenotypically complete or mosaic albinos. Molecular genotyping identified three mice with 9.5 kb deletions and upon breeding two of these transmitted this deletion to the next generation (Table 1). Sequencing of genomic DNA from the albino and mosaic albino mice across the gRNA cut sides identified significant allelic heterogeneity in each mouse resulting from the error‐prone non‐homologous end joining (Fig. 1). The second allele in mice with the deletion had small insertions and/or deletions at the CRISPR/Cas9 cut sites in either exon 1 or exon 2. The number of different alleles identified in each mouse varied from 1 to 4 and none of the mice carrying the deletion had incorporated the bridging oligonucleotide.

**Table 1:** Efficiencies of Generating and Transmitting Small Deletions

	Injections and births		G0 mice with			
Deletion size	Embryos transferred	Pups born (%)	Albino/mosaic coat (%)	Imprecise deletion (%)	Precise deletion (%)	Germ line transmission (%)
9,376 bp	100	30 (30%)	10 (33%)	3 (10%)	0 (0%)	2 (67%)
64,770 bp	224	81 (36%)	31 (38%)	10 (12%)	3 (4%)	7 (54%)

We next extended the study to a 65 kb deletion covering the whole albino locus (Fig. 1). Given that there appeared to be significant cutting and re‐joining of the break‐point junctions for the 9.5 kb interval which did not resolve into a structural variant, we reasoned that targeting additional double strand breaks at each endpoint would elevate the frequency of generating this class of allele. Four gRNAs were co‐injected which targeted the first and last *Tyr* exons along with a bridge oligonucleotide. Out of the 81 founders born, 38% were albino or mosaic albino, 10 (12%) of these carried an imprecise deletion, and 3 (4%) had correctly incorporated the bridging oligonucleotide in the repair process (Table 1; Fig. 1). Upon breeding, 7 of these transmitted the deletion to the next generation (54%).

### Generating Large Structural Variants Around the Try Locus

**Figure 2:**
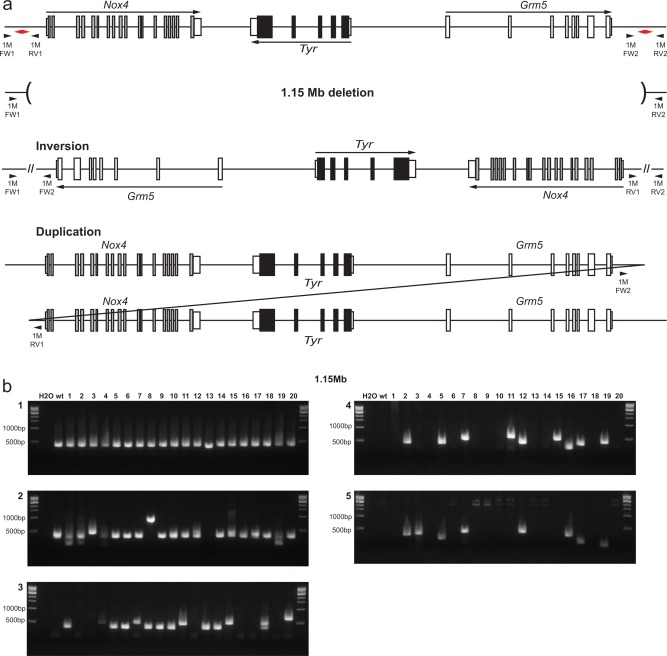
Generation of large structural variants. (**a**) Schematic representation of the possible outcomes of genomic rearrangements. gRNA sites are indicated with *red arrowheads*, genotyping primer sites with *black arrowheads*. (**b**) PCR genotyping of DNA from 20 founder mice for the 1.15 Mb genomic rearrangements. Panels 1 and 2, analysis of the breakpoints using primer sets which flank the gRNA cut sites at the proximal and distal ends of the region. Panel 3, deletion breakpoint analysis with FW1 + RV2 primers. Panels 4 and 5, inversion breakpoint analysis at the proximal (FW1 + FW1) and distal (RV1 + RV2) ends of the inversion, respectively.

Several junction points in the proximity of the *Tyr* locus were selected to explore the feasibility and assess the limits of generating larger rearrangements in the 0.1–1.0 Mb range (Fig. 2). Two pairs of gRNAs were used for each study, which were co‐injected with the corresponding bridge oligonucleotides. The gRNA pairs were used in combination to delete individual genes, *Nox4* (155 kb) and *Grm5* (545 kb) as well as the whole region from *Nox4* to *Grm5* including *Tyr* (1.15 Mb) (Supporting Information Fig. 2a).

More than half of the injected embryos developed to term. The resultant founder mice were genotyped for the full spectrum of variants, using a variety of polymerase chain reaction (PCR) assays (Fig. 2; Supporting Information Fig. 2). This revealed that nearly half of the pups born possessed one of the desired structural variants (Table 2). Mice with variants were identified at similar frequencies for each of the interval tested. The variants detected were either deletions or inversions in equal numbers. Mice with duplications were identified from the 155 and 545 kb intervals but these were infrequent and no examples were identified for the 1.15 Mb interval (Table 2).

**Table 2:** Efficiencies of Generating Large Rearrangements

	Injections and births		G0 mice with			
Deletion size	Embryos transferred	Pups born (%)	Imprecise deletion (%)	Precise deletion (%)	Inversion (%)	Duplication (%)
1,55,288 bp	105	46 (44%)	7 (15%)	4 (9%)	14 (30%)	1 (2%)
5,45,426 bp	114	68 (60%)	10 (15%)	2 (3%)	12 (18%)	1 (1%)
1,151,853 bp	103	48 (47%)	6 (13%)	8 (17%)	10 (21%)	0 (0%)

Allelic heterogeneity was also observed. Given the loss of sequence at some of the breakpoints, some inversions were scored with one breakpoint only. Many of the founders were mosaics (Supporting Information Table 1). Mice could be identified with both an inversion as well as a deletion allele, others were observed with wild type, inversion and deletion alleles, and yet others were mosaic with an inversion, a deletion, and indels which could be detected at one or both breakpoints on a non‐rearranged chromosome. Sequencing of DNA samples from the mice revealed the expected allelic heterogeneity at the breakpoints observed in the outcome of the experiments with the 9.4 and 64.8 kb deletions. The co‐injected oligonucleotide led to the generation of mice with a defined deletion for all of the intervals tested at an overall rate of 9%.

Upon breeding at least 50% transmitted the deletion or inversion to the next generation independent of the rearrangement size (Supporting Information Table 2). Fluorescent in situ hybridization was conducted on some of the F1 pups which confirmed the rearrangements detected by PCR analysis (Supporting Information Fig. 3).

To further understand the contribution of the different gRNAs in the generation of the rearrangements we determined the cutting efficiency of each gRNA by analysis of the INDEL rate. The allele structure in the founder mice is complex due to mosaicism; therefore, we breed the founders to segregate their chromosomes and examined the gRNA target sites in mice which did not carry rearrangements (Supporting Information Table 3). INDEL efficiencies ranging from 8% to 90% were observed. In cases where cutting is highly efficient, for instance Grm5‐3 in which 18 out of 19 mice carried an INDEL, we observed additional INDELS on the same chromosome generated by the co‐injected gRNAs. In some cases up to three sites on the same chromosome have INDELs. Thus, even in cases where cutting is highly efficient, this frequently does not resolve in a rearrangement.

We have examined the 1.15 Mb inversion breakpoints to identify which gRNA sites are responsible for the inversion breakpoints. On one side in 3 out of 10 cases the inversion utilized the outer gRNA with an INDEL efficiency of 14% rather than the adjacent gRNA with an INDEL efficiency of 90% (Supporting Information Fig. 4). We find a similar situation on the other breakpoints. On this basis we can conclude that different combinations of gRNAs contribute to the generation of the inversions. Moreover, although the more efficient gRNA was used most frequently, this was not saturating because some inversions were generated by the less efficient gRNA.

## DISCUSSION

The CRISPR/Cas9 system is a very efficient and comparatively fast method to generate genome edited mice with small modifications such as insertion–deletions of a few nucleotides and the introduction of short tags via direct injection in zygotes (Wang *et al*., 2013; Yang *et al*., 2013, 2014). Here we extend this technology to very large chromosomal rearrangements, facilitating the facile generation of mouse models with this important class of structural variants.

Cytoplasmic injection of mouse zygotes with spCas9 mRNA, two pairs of gRNAs directed to the desired breakpoints and an oligonucleotide which bridges the breakpoints was used to generate these variants. Founder mice harboring a desired structural variant such as a deletion or an inversion of up to 1.15 Mb could be generated at rates of up to 30%. Although duplications can also be generated these are detected at much lower frequencies. Structural re‐rearrangements in this study were identified using PCR assays for specific junction fragments which may not identify all mice with rearrangements. The detection of inversion at one, rather than two breakpoints, demonstrates that there are false negatives (Supporting Information Table 1). PCR amplification of a breakpoint requires that the primer sites are retained at the junctions, which may in some cases be degraded beyond the primer binding sites. In many cases the founder mice were mosaic, most easily detected from the observation of small patches of albino hair in otherwise black mice. This is reminiscent of chimaeras resulting from ES cell injection at the blastocyst stage (3.5 days). Given the limited number of DNA target molecules available and the vast excess of both Cas9 and gRNA, cutting and re‐joining must occur for an extended period of time, until the site is destroyed by an INDEL or a structural variant. Analysis of somatic DNA from the ear and germ line transmission detects up to 4–5 alleles which suggests that Cas9 is active to the 2–4 cell stage at least.

The generation of an inversion or deletion requires two break and re‐joining events on the same chromosome, which using dual gRNAs is efficiently achieved. The occurrence of combinations of deletions and inversions in single founder mice, often with mosaicism with an unmodified and/or homologue, illustrates that the overall efficiency of the process is high. To generate founder mice with this genotype, the CRISPR/Cas9 process must be active on either homologues or two sister chromatids simultaneously and/or cutting and re‐joining may continue after the first zygotic division. We reasoned that the provision of two pairs of gRNAs targeted to each breakpoint, would extend the temporal window for achieving the desired modification by providing the opportunity for two complete cycles of error‐prone repair before the target sites are destroyed. Analysis of the resultant mice confirms that different combinations of the gRNAs are able to contribute to the generation of the structural variant, not just the most efficient ones. In some cases variants are found in which the less efficient gRNA has been used to generate the break point, but the same chromosome contains an INDEL generated by the more efficient gRNA. We cannot resolve if the INDEL was generated before or after the re‐arrangement, but it is not unreasonable to conclude that because of its efficiency its target site was rapidly destroyed by INDEL formation thereby excluding this gRNA from contributing to the process. We can further conclude that although two gRNAs are sufficient more events are generated with four.

In these experiments we sought to generate deletions with nucleotide precision through the provision of a repair template in the form of a single strand oligonucleotide. The sequence of the oligonucleotide was designed to omit the Cas9 cleavage sites to prevent re‐cleavage of the repaired chromosome by continued CRISPR/Cas9 cutting. Precise deletion junctions were successfully generated for all but one of the intervals attempted in this study, although this was most efficient for the 1 Mb deletion. While we do not understand the reason for this, the proximity of the broken ends may favor simple NHEJ for smaller genetic intervals. The oligonucleotide directed the repair of the break in 17 out of 53 (32%) of the mice born. One potential reason for this is exonuclease activity may resect the broken DNA at the break point beyond the limit of the sequence in the oligonucleotide. Overall, an equal number of deletions (precise plus imprecise) and inversions were generated (Table 2). Thus, although the bridging oligonucleotide allows deletions to resolve with precise junctions, it does not appear to stimulate more events, suggesting that the cutting efficiency is rate limiting.

The infrequent generation of duplications, reflects the fact that this is a significantly more complex event to achieve than either a deletion or an inversion. Duplications require three chromosomal breaks, two flanking the duplicated region, and a third at the integration site. Furthermore, several competing pathways are available to the excised fragment of DNA only one of which results in a duplication. Most often the fragment may re‐integrate back into its original site generating a chromosome with two NHEJ events at the breakpoints with the excised piece in the original or an inverted orientation. Duplications can only be generated if two copies of the genome are available in the same nucleus, one providing the donor sequence and the second the integration site. In the context of a zygote, in which the pro‐nuclei are separate compartments, two copies only become available as sister chromatids after DNA replication or after pronuclear fusion which unites the homologous chromosomes. This places significant temporal constraints on the process, given that homologous chromosomes reside in separate male and female pro‐nuclei at the time of CRISPR/Cas9 microinjection and for several hours afterward. Although we cannot resolve which pathway is favored in these experiments, it is possible that the efficiency of generating duplications could be enhanced by injecting the CRISPR/Cas9 components just prior to pro‐nuclear fusion.

Recently Kraft *et al*. (2015) described the generation of structural variants using CRISPR/Cas9 in ES cells including the successful transmission of one allele. The efficient generation of these variants directly in zygotes in our study significantly expands their availability and broadens their utility. Structural variants can in principle be engineered in any mouse line (or in other species where ES cells do not exist) and these variants can be directly introduced into mouse lines with existing combinations of alleles, a process which would otherwise require substantial breeding if the starting point was a previously unmodified ES cell. The efficiency of generating even very large deletions suggests this method will replace ES‐cell based methods of chromosome engineering.

## MATERIALS AND METHODS

### CRISPR/Cas9 Target Sites and Vector Construction

CRISPR/Cas9 target sites were identified using http://crispr.mit.edu/as described (Ran *et al*., 2013). In addition the guide RNAs were picked following the guidelines from Doench *et al*. (2014) avoiding C and T upstream of the PAM, G downstream of the PAM and T within the PAM whenever possible. For the deletion of the 9.5 kb region, single gRNAs positioned either side of the deletion endpoints on opposite strands were selected (Supporting Information Table 2). For all other deletions, pairs of gRNAs were designed for each endpoint. These were typically located within 50–200 bp of each other and positioned on opposite strands (Supporting Information Table 4). Single strand oligonucleotides (ssODN) designed to bridge the deletions were 120 bp in length and positioned directly adjacent to the most external gRNA site.

The gRNA oligonucleotides were synthesized, the two strands annealed and cloned into a vector containing the gRNA backbone and a T7 promoter for RNA production using *BsaI*. For Cas9 mRNA production the vector from Zhang (Cong *et al*., 2013) was modified to contain the T7 promoter. The integrity of all plasmids was confirmed by DNA sequencing. The ssODN were synthesized by Integrated DNA Technologies (IDT).

### Cas9 and gRNA Production

For Cas9 RNA production, the T7/Cas9 plasmid was linearized with *EcoRI* and for gRNA production with *DraI*. The plasmids were cleaned with a PCR purification kit (Quiagen) and in vitro transcribed using mMessage mMachine T7 Ultra kit and MEGAshortscript T7 kits (Life Technologies), respectively. Both, Cas9 mRNA and gRNA, were purified using the MEGAclear kit (Life Technologies) and eluted in RNase‐free water. The quality of the RNA was analyzed using Agilent RNA 6000 Nano kit (Agilent Technologies, 2100 Bioanalyzer) and Qubit RNA HS assay kit (Life Technologies). The bridging oligonucleotides were dissolved in RNAse‐free water to a concentration of 1,000 ng/μL.

### Zygote Injection

Four‐ to five‐week‐old C57BL/6NTac females were super‐ovulated by intraperitoneal (IP) injection of 5 IU of pregnant mare's serum (PMSG) at 12:00 to 13.00 hours (on a 12 hour light/dark cycle, on at 07:00/off at 19:00) followed 48 hours later by an IP injection of 5 IU human chorionic gonadotrophin (hCG) and mated overnight with C57BL/6NTac stud males. The next morning the females were checked for the presence of a vaginal copulation plug as evidence of successful mating, oviducts were dissected at approximately 21–22 hours post HCG and cumulus oocyte complexes from these were released and treated with hyaluronidase as previously described (Behringer *et al*., 2014). Fertilized 1‐cell embryos were selected and maintained at 37°C in KSOM media prior to cytoplasmic injection. Injections were carried out between 24 and 27 hours post HCG.

About 50 ng/μL Cas9 mRNA, 25 ng/μL gRNA (each) and 100 ng/μL oligonucleotide were mixed in RNase free water, backfilled into an injection needle with positive balancing pressure and injected into the cytoplasm of fertilized 1‐cell embryos held in FHM medium. A successful injection was indicated by visible movement in the cytoplasm (Supporting Information Fig. 1A). Injected embryos were briefly cultured and viable embryos were transferred the same day by oviducal embryo transfer into a 0.5 days post coital pseudo‐pregnant female F1 (CBA/C57BL/6J) recipients (Behringer *et al*., 2014).

All procedures performed in studies involving animals were in accordance with the ethical standards of the institution or practice at which the studies were conducted and performed with approval of the UK home office.

### DNA Isolation and Genotyping of Mutant Founders and Their Offspring

Genomic DNA was isolated from ear clips of founder mice and their offspring. About 1 μL of the earclip lysate was used per PCR reaction with High Fidelity Platinum Taq polymerase (Life Technologies). The PCR products were examined on gels and sequenced to ascertain the integrity of inversions, duplications, and deletions. Resulting positive F0 founders were bred to examine transmission of the detected alterations (Table 1 and Supporting Information Table 2).

### Fluorescent In Situ Hybridization

For the deletion, B lymphocytes isolated from spleens were grown in RPMI + 10% FBS, stimulated using lipopolysaccharide (LPS, Sigma‐Aldrich) and after 44 hours blocked in metaphase with 0.1 μg/mL KaryoMax Colcemid (Gibco Life Technologies) for 2 hours prior to harvesting chromosomes. These were hybridized with probes using a standard FISH protocol (Liu *et al*., 2013). The following BAC (Bacterial artificial chromosome) clones were use: RP23‐63G7 (87,292,751–87,445,311) labeled with Aminoallyl‐dUTP‐Texas Red (Jena‐Bioscience), RP24‐102H24 (87,884,864–88,075,187) labeled with Aminoallyl‐dUTP‐XX‐Atto‐488 (Jena‐Bioscience) within the genome edited region. RP23‐335D4 (88,499,299–88,687,302) labeled with Aminoallyl‐dUTP‐Cy3 (Jena‐Bioscience) was used as the control probe outside the genome edited region (Supporting Information Table 6).

For analysis of inversions, non‐cultured spleen cells were used. The following BAC clones were used; RP23‐63G7 (87,292,751–87,445,311) labeled with Aminoallyl‐dUTP‐Texas Red and RP24‐102H24 (87,884,864–88,075,187) Aminoallyl‐dUTP‐Cy5 (Jena‐Bioscience) within the genome edited region. The control probes RP23‐84N6 (86,864,511–87,112,279) labeled with Aminoallyl‐dUTP‐XX‐Atto‐488 and RP23‐335D4 (88,499,299–88,687,302) labeled with Aminoallyl‐dUTP‐Cy3, lying outside the genome edited region (Supporting Information Table 6).

The BAC clones were prepared with a BAC DNA kit (Sigma‐Aldrich), amplified using a whole genome amplification kit (WGA‐2, Sigma‐Aldrich). Using 10–100 ng of purified genomic BAC DNA the WGA‐2 products were then directly labeled using a GenomePlex Reamplification kit (WGA‐3, Sigma) with Aminoallyl‐dUTP‐Cy3, Aminoallyl‐dUTP‐Cy5, Aminoallyl‐dUTP‐Texas Red, and Aminoallyl‐dUTP‐XX‐Atto‐488, respectively. Hybridization and washes were carried out by standard procedures.

FISH images were captured and processed using the SmartCapture (Digital Scientific, The United Kingdom) digital imaging system using a Zeiss microscope (Axioplan 2 Imaging or AxioImager, DI) equipped with narrow bandpass filters for Cy5, Cy3, Texas Red, FITC and DAPI fluorescence, a cooled CCD camera (Hamamatsu).

## AUTHOR CONTRIBUTIONS

KB designed and performed most experiments. BD developed and performed the cytoplasmic injections. RB and FY designed and performed the fluorescent insitu hybridization. AB devised and coordinated the project, and together with KB, designed experiments, interpreted data and wrote the manuscript.

## Supporting information

Supporting Information Figure 1.

Supporting Information Figure 2.

Supporting Information Figure 3.

Supporting Information Figure 4.

Supporting Information Tables.
