# Supplementary material for: Chromosome engineering in zygotes with CRISPR/Cas9
Source: Genesis. 2016 Jan 25;54(2):78–85. doi: 10.1002/dvg.22915 (PMC4819711; doi:10.1002/dvg.22915)
Supplement: Supplementary file 5 — Supporting Information Tables. [file DVG-54-78-s005.doc]

Supplementary tables

| **Extended Table 1: Efficiencies for the different variations of genomic rearrangements** | | | |
| --- | --- | --- | --- |
|  | **Design size** | | |
| **Different genomic rearrangements** | **155,288bp** | **545,426bp** | **1,151,853bp** |
| Deletion | 7 (15%) | 7 (10%) | 9 (19%) |
| Duplication | 1 (2%) | 1 (1%) | 0 (0%) |
| Inversion (both breakpoint PCRs positive) | 2 (4%) | 6 (9%) | 5 (10%) |
| Inversion (one breakpoint PCR positive) | 6 (13%) | 4 (6%) | 1 (2%) |
| Inversion or deletion with larger indel on 2nd allele | 2 (4%) | 3 (4%) | 1 (2%) |
| Deletion and Inversion | 1 (2%) | 0 (0%) | 0 (0%) |
| Mosaic: one allele wt/indel, one allele deletion, one allele inversion | 3 (7%) | 2 (3%) | 4 (8%) |

| **Extended Table 2: Germ Line Transmission (GLT) rates of large rearrangements** | | | | | |
| --- | --- | --- | --- | --- | --- |
| **size** | **Rearrangement** | **# bred** | **# GLT(%)** | **germ line mosaicism** | **germ line average** |
| **155,288bp** | Deletion | 6 | 3 (50%) | 0-86% | 32% |
|  | Inversion | 10 | 6 (60%) | 0-63% | 32% |
|  | Deletion+Inversion Del GLT only | 4 | 2* (50%) | 43-67% | 55% |
|  | Deletion+Inversion Inv GLT only |  | 2* (50%) | 67-75% | 71% |
|  | Deletion+Inversion BOTH GLT |  | 2* (50%) | 67-75% | 71% |
|  | Duplication | 1 | 0 (0%) | NA | NA |
| **545,426bp** | Deletion | 10 | 8 (80%) | 0-63% | 35% |
|  | Inversion | 8 | 5 (63%) | 0-100% | 36% |
|  | Deletion+Inversion Del GLT only | 2 | 1 (50%) | 88% | NA |
|  | Deletion+Inversion Inv GLT only |  | 1 (50%) | 63% | NA |
|  | Deletion+Inversion BOTH GLT |  | 0 (0%) | NA | NA |
|  | Duplication | 1 | 0 (0%) | NA | NA |
| **1,151,853bp** | Deletion | 10 | 8 (80%) | 0-100% | 44% |
|  | Inversion | 6 | 3 (50%) | 0-38% | 18% |
|  | Deletion+Inversion Del GLT only | 4 | 2 (50%) | 25-50% | 38% |
|  | Deletion+Inversion Inv GLT only |  | 2 (50%) | 40-44% | 43% |
|  | Deletion+Inversion BOTH GLT |  | 0 (0%) | NA | NA |
| * some of the offspring only contained a deletion or inversion whereas others contained both | | | | | |

| **Extended Table 3: cutting efficiencies of gRNAs** | | |
| --- | --- | --- |
| **gRNA** | **# of sequences analysed** | **indel efficiency (%)** |
| **Nox4 1** | 24 | 2 (8%) |
| **Nox4 2** | 24 | 6 (25%) |
| **Nox4 3** | 24 | 10 (42%) |
| **Nox4 4** | 24 | 7 (29%) |
| **Grm5 1** | 21 | 8 (38%) |
| **Grm5 2** | 21 | 6 (29%) |
| **Grm5 3** | 19 | 18 (95%) |
| **Grm5 4** | 21 | 4 (19%) |
| **1M 1** | 21 | 4 (19%) |
| **1M 2** | 21 | 9 (43%) |
| **1M 3** | 22 | 3 (14%) |
| **1M 4** | 20 | 18 (90%) |

| **Extended Table 4: Guide RNA and oligo sequences for the different deletions** | | | | | | | |
| --- | --- | --- | --- | --- | --- | --- | --- |
| **deletion** | **gRNA** | | **sequence (5' - 3')** | | **coordinates** | **oligo sequence (5' - 3')** | |
| **9.5kb** | **Tyr 1** | | TGCCAACAAGTTCTTAGAGGAGG | | 7:87,493,253-87,493,278 | TTTCTAAAGCTGAAATTGGCAGTTCTATCCATTGATCCAGATTCATACTGGGTCAAACTCAGACAAAATTATCGATCACAGGCTCGAGGAAAATGGCCATCAGAGATCTGGAAACTCCACAGAAGGCAATACAAAACAGCCAAGAACATTTTCTCC | |
|  | **Tyr 2** | | GCTCCCATCTTCA­GCAGATGTGG | | 7:87,483,900-87,483,925 |  |  |
| **65kb** | **Tyr 3** | | TGTGACACTCATTAACCTATTGG | | [7:87,493,380-87,493,402](http://www.ensembl.org/Mus_musculus/Location/View?r=7:87493378-87493403;tl=Vlu1OhQOcthGfZIy-1041408-218283225) | AAAACAAAAATGTGCCTCAAGTTTAAATAATTAAGACACATTCATAAAAACGATCGTAACTAAATTGAAGGAAGATATATTATTCTAATTGATATGAAATTAATAATAATTGGAAT | |
|  | **Tyr 4** | | TTTGTACATAGCCCATAGTGAGG | | [7:87,493,552-87,493,574](http://www.ensembl.org/Mus_musculus/Location/View?r=7:87493550-87493575;tl=Vlu1OhQOcthGfZIy-1041409-218283199) |  |  |
|  | **Tyr 5** | | ACCAACAAATAGGTCGAGTGAGG | | [7:87,428,990-87,429,012](http://www.ensembl.org/Mus_musculus/Location/View?r=7:87428988-87429013;tl=Vlu1OhQOcthGfZIy-1041410-218283207) |  |  |
|  | **Tyr 6** | | CCTCTTACAACTAATTGAGCTGG | | [7:87,428,805-87,428,827](http://www.ensembl.org/Mus_musculus/Location/View?r=7:87428803-87428828;tl=Vlu1OhQOcthGfZIy-1041411-218283208) |  |  |
| **155kb** | **Nox4 1** | | AAAACTCCAAGTTGGGATGCAGG | | [7:87,245,205-87,245,227](http://www.ensembl.org/Mus_musculus/Location/View?r=7:87245203-87245228;tl=nnfadwBDe1dTXmfp-1041413-218283340) | CAACCTGGTCTTCATGTGGGTTCCAAAAATTTGGAGTGAAGGCTATCCCAAAAGCTGTTGGCGGCCGCAGATTTGCATCTGAGAAGTTATCTGCTGAACTTGCTCCTCCTATCCTGGA | |
|  | **Nox4 2** | | CAGCCTCTCATTTGGGGCATGGG | | [7:87,245,248-87,245,270](http://www.ensembl.org/Mus_musculus/Location/View?r=7:87245246-87245271;tl=nnfadwBDe1dTXmfp-1041414-218283329) |  |  |
|  | **Nox4 3** | | GCTCCGAGGAAAAGTCGAGGGGG | | [7:87,400,470-87,400,492](http://www.ensembl.org/Mus_musculus/Location/View?r=7:87400468-87400493;tl=nnfadwBDe1dTXmfp-1041416-218283349) |  |  |
|  | **Nox4 4** | | GACATATCCCTTATATATACAGG | | [7:87,400,344-87,400,366](http://www.ensembl.org/Mus_musculus/Location/View?r=7:87400342-87400367;tl=nnfadwBDe1dTXmfp-1041415-218283336) |  |  |
| **545kb** | **Grm5 1** | | GGGGACTATCGATGTATGCAGGG | | [7:87,597,333-87,597,355](http://www.ensembl.org/Mus_musculus/Location/View?r=7:87597331-87597356;tl=EzGGHl1CjnHLMGNc-1041432-218283503) | GAGTTCCATAACAGGGTGTTGCTTGCTTAGAAGTAAGTGGGGGCTCCTTAAGTGCCAGAGGCGGCCGCAAATTAAGCAGAAATGTAACTCAGCTTACAAACCTCACAGAAAAATATCA | |
|  | **Grm5 2** | | CACACAAACATATTATAGTTTGG | | [7:87,597,304-87,597,326](http://www.ensembl.org/Mus_musculus/Location/View?r=7:87597302-87597327;tl=EzGGHl1CjnHLMGNc-1041433-218283506) |  |  |
|  | **Grm5 3** | | AACTGTTTGTGCTATCCATTGGG | | [7:88,142,707-88,142,729](http://www.ensembl.org/Mus_musculus/Location/View?r=7:88142705-88142730;tl=EzGGHl1CjnHLMGNc-1041434-218283476) |  |  |
|  | **Grm5 4** | | TCATACCACCTTTTACTCCTGGG | | [7:88,142,588-88,142,610](http://www.ensembl.org/Mus_musculus/Location/View?r=7:88142586-88142611;tl=EzGGHl1CjnHLMGNc-1041435-218283484) |  |  |
| **1.15Mb** | **1M 1** | | CCTGATTTATGTTTCGTACGTGG | | [7:87,078,672-87,078,694](http://www.ensembl.org/Mus_musculus/Location/View?r=7:87078670-87078695;tl=3aMdXx9BO3T0iys7-1041439-218283523) | GATGACATGCAGAAATTAGAGGACAACTTACCACAGTTAGTTCTCTTCTACTATGTGTGTGCGGCCGCTTTAGTACAGAGTTCTATCAGACCTTCAAAGAAGATCTAATTCCAGTTCT | |
|  | **1M 2** | | AGCCCAAGACCAGACCAGATGGG | | [7:87,078,562-87,078,584](http://www.ensembl.org/Mus_musculus/Location/View?r=7:87078560-87078585;tl=3aMdXx9BO3T0iys7-1041440-218283526) |  |  |
|  | **1M 3** | | CTGTGCACATGGGCGCAAACAGG | | [7:88,230,281-88,230,303](http://www.ensembl.org/Mus_musculus/Location/View?r=7:88230279-88230304;tl=3aMdXx9BO3T0iys7-1041441-218283517) |  |  |
|  | **1M 4** | | CAACCTGAGTCCAGTCCTTGGGG | | [7:88,230,392-88,230,414](http://www.ensembl.org/Mus_musculus/Location/View?r=7:88230390-88230415;tl=3aMdXx9BO3T0iys7-1041442-218283536) |  |  |
| All sequences are from 5' to 3'; PAMs are underlined. | | | | | | | |
| **Extended Table 5: Genotyping primer sequences for the different rearrangements** | | | | | | | |
| **deletion** | | **primer** | | **sequence (5' - 3')** | | | **coordinates** |
| **9.5kb** | | **Tyr FW1** | | AGGAACCTCTGCCTGAAAGC | | | 7:87,493,188-87,493,207 |
|  |  | **Tyr RV1** | | AACTGCCAGAAAGCTGAATGA | | | 7:87,484,110-87,484,130 |
| **65kb** | | **Tyr FW2** | | AGGAAAACAAAAATGTGCCTCA | | | 7:87,428,752-87,428,773 |
|  |  | **Tyr RV2** | | TCAGAATCTAGATGTTTCATGACCT | | | 7:87,493,682-87,493,706 |
| **155kb** | | **Nox4 FW1** | | AACTCAAAATAGTTTCCTGGACATC | | | 7:87,245,081-87,245,105 |
|  |  | **Nox4 RV1** | | TCTCAAGCATGAGTGAGTCAAGA | | | 7:87,245,492-87,245,514 |
|  |  | **Nox4 FW2** | | AAAGGACCCTGCTCAAGACTC | | | 7:87,400,142-87,400,162 |
|  |  | **Nox4 RV2** | | AGAGTCTGAACACCCAACCA | | | 7:87,400,605-87,400,624 |
| **545kb** | | **Grm5 FW1** | | TTTGGTCCAACCATCCACTCAA | | | 7:87,597,126-87,597,147 |
|  |  | **Grm5 RV1** | | AATCTGGGCATGACCTCTCA | | | 7:87,597,521-87,597,540 |
|  |  | **Grm5 FW2** | | ACATTTATCCAGTCCAGGGGTG | | | 7:88,142,481-88,142,502 |
|  |  | **Grm5 RV2** | | AATGCCAACTATGCCTTTGCT | | | 7:88,142,811-88,142,831 |
| **1.15Mb** | | **1M FW1** | | TGTGTTCTTGGATTCAGTTAGCG | | | 7:87,078,324-87,078,346 |
|  |  | **1M RV1** | | TCAGATCCTTCTACCAAAGGCT | | | 7:87,078,749-87,078,770 |
|  |  | **1M FW2** | | GCAACTGGTGAAGTACCGAGA | | | 7:88,230,191-88,230,211 |
|  |  | **1M RV2** | | CGTGTTCCCTATCTGGCCTT | | | 7:88,230,557-88,230,576 |

| **Extended Table 6: Probes used for FISH analysis** | | | |
| --- | --- | --- | --- |
| **rearrangement** | **probe** | **coordinates** | **colour** |
| **1.15Mb deletion** | RP23-63G7 | 7: 87,292,751-87,445,311 | red |
|  | RP24-102H24 | 7: 87,884,864-88,075,187 | green |
|  | RP23-335D4 | 7: 88,499,299-88,687,302 | yellow |
| **1.15Mb inversion** | RP23-84N6 | 7: 86,864,511-87,112,279 | green |
|  | RP23-63G7 | 7: 87,292,751-87,445,311 | red |
|  | RP24-102H24 | 7: 87,884,864-88,075,187 | pink |
|  | RP23-335D4 | 7: 88,499,299-88,687,302 | yellow |

## Supplementary Figure Legends

### Extended Data Figure 1: Cytoplasmic injection and coat colour

a) Schematic representation of cytoplasmic injections of the CRISPR/Cas9 material into C57BL6/N zygotes.

b) Different outcomes of the coat colours after CRISPR/Cas9 deletions of *Tyrosinase*. Heterozygous mutation/deletion of *Tyrosinase* results in black coat colour whereas homozygous deletion results in albino patches or complete albinism.

### Extended Data Figure 2: Generation of large deletion using CRISPR/Cas9

a) Schematic representation of the CRISPR/Cas9 gRNA sites (red arrowheads) for the 155kb, 545kb and 1.15Mb deletions as well as genotyping primer sites (black arrowheads).

b) PCR genotyping of DNA samples from 22 founder mice for the 155 kb (*Nox4*) and 23 founder mice for 545 kb (*Grm5*) genomic rearrangements. Panels 1-6 show the results of the analysis of the 155 kb interval. Panels 7-12 show the analysis of the 545 kb interval. 1 and 2, break point analysis of the 5’ and 3’ ends of the *Nox4* gene respectively using primers which flank the gRNA targets sites. 7 and 8, break point analysis of the 5’ and 3’ ends respectively of the *Grm5* gene. 3 and 9, deletion junction analysis using FW1 and RV2 primer sets for the *Nox4* and *Grm5* loci. 4 and 10, inversion junction analysis using FW1 + FW2 primer sets at the 5’ end of the *Nox4* and *Grm5* loci respectively. 5 and 11, inversion junction analysis using RV1 + RV2 primer sets at the 3’ end of the *Nox4* and *Grm5* loci respectively. 6 and 12, duplication junction analysis using primer sets FW2 + RV1.

### Extended Data Figure 3: FISH analysis of the 1.15Mb rearrangements

a) For detection of deletions, 3 probes were designed. Two were internal to the expected deletion (red and green) and one probe was external of the expected deletion and should thus be unaffected. Metaphase cells of spleens from F1 generation pups were used for this analysis. Wild type alleles should show a red, green and yellow pattern whereas a deletion allele should show only the yellow probe (red arrow).

b) For detection of inversions, 4 probes were designed. Two probes were internal (red and pink) and two probes were external either side of the expected inversion (green on the 5’ end and yellow on the 3’end). Interphase cells of spleens from F1 generation pups were used for this analysis. Wild type alleles should show the green and red probes close to each other on the 5’end and pink and yellow being close to each other on the 3’end. Inversions will show the green probe being close to the pink one on the 5’ end and the yellow probe being close to the red probe (red arrow).

**Extended Data Figure 4: Sequences of large inversions generated by CRISPR/Cas9**

PCR products of the 10 founders carrying inversions were sequenced and show that even gRNAs with low efficiencies, such as 1M 3 contributed to the generation of inversions.
